# Using appreciative inquiry to transform student nurses’ image of nursing

**DOI:** 10.4102/curationis.v38i1.1460

**Published:** 2015-08-19

**Authors:** Motshedisi E. Chauke, Dirk Van Der Wal, Annalie Botha

**Affiliations:** 1 Department of Health Studies, University of South Africa, South Africa

## Abstract

**Introduction:**

Literature provides adequate evidence of a poor perception of nursing within the profession, resulting in high rates of attrition of student nurses and newly qualified nurses. The nursing profession, in particular nurse educators, has an ethical and professional responsibility to find innovative strategies to promote the positive image of nursing amongst student nurses.

**Purpose:**

The purpose of the study was to explore the potential of appreciative inquiry (AI) as an intervention teaching strategy to transform student nurses’ image of nursing.

**Design:**

A quantitative, quasi-experimental, explorative-descriptive design comprising the pretest, appreciative inquiry as intervention, and the post-test was used.

**Methods:**

Convenience sampling was used to select third and fourth year college and university student nurses in the Gauteng province of South Africa for the pre- and the post-test respectively. Data were collected by means of a questionnaire and analysed by SPSS version 20.0.

**Findings:**

The pretest results revealed a mix of positive and negative perceptions of the image of nursing amongst student nurses. The negative perceptions of the image of nursing that needed intervention included the working conditions of nurses, and the perception of nursing as a profession that was not respected and appreciated. The post-test results showed a significant and positive change in the student nurses’ perception of the image of nursing as a respected and appreciated profession. Although AI resulted in a negative to positive change in some aspects of student nurses’ image of nursing, the negative perceptions of the working conditions of nurses remained and became more negative. The positive image of gender in nursing was enhanced following the implementation of AI.

**Conclusion:**

Appreciative inquiry demonstrated potential as a teaching strategy to produce a positive nursing image change and positive orientation towards nursing amongst student nurses.

## Introduction

A plethora of different studies reveals that the nursing profession is faced with image-related challenges impacting on its status, prestige and the ability to attract more young, and suitably qualified, students to nursing and to retain students in nursing programmes (Breirer, Wildschut & qolozana 2009:121; Hoke 2006:94; Mkhize & Nzimande 2007:17; Mphahlele 2011:11; Pillay 2009; Regan 2005:210; South Africa 2008:10; Van Zyl 2011:12). The literature also reveals that, in spite of the use of many professional socialisation strategies, there is evidence of negative perceptions of nursing amongst student nurses resulting in high rates of attrition of student nurses and newly qualified nurses. The challenges of the poor image of nursing, high rates of attrition of student nurses and newly qualified nurses were acknowledged in the Department of Health document, Nursing Strategy for South Africa (2008); they were also acknowledged in the Nursing Compact (South Africa 2012), which was drafted by the nurses at the South African (SA) National Nursing summit in 2011. These issues pose a serious threat to the future of the nursing profession and necessitate a need for innovative strategies to promote a positive professional self-image and positive orientation towards nursing amongst student nurses (Darbyshire & Gordon 2005:74; Natan 2009:9; Palumbo *et al.* 2008:9; Parvan, Zamanzadeh & Hosseini 2012; South Africa 2008:11; South Africa 2012:77).

For many students nursing was not the first career choice (Kiwanuka 2010:15; Mkhize & Nzimande 2007:14). It was only considered when they could not be accepted into other courses such as medicine (Neilson & Lauder 2008:687). The findings of studies by Breier *et al.* (2009:75) and Mkhize and Nzimande (2007:22) show that many SA students entered nursing degree programmes because these offered the opportunity to earn whilst studying and not because they considered it as a career option of choice. According to Breier *et al*. (2009:124), some of the students did not seem to be motivated to become nurses at the end of their studies and, in some cases, even to complete their studies, resulting in high rates of attrition of student nurses and newly qualified nurses. However, others grew to love and appreciate nursing as a career because they had gained a better understanding of the profession. Such positive perceptions of nursing amongst student nurses are important for the image of nursing and should be nurtured and sustained.

The students for whom nursing was the first choice of career entered nursing with a perception of nursing as caring and nurturing that requires special characteristics such as patience, empathy and emotional strength. But some of these students were disappointed and disillusioned by what they experienced during their training, hence the large scale dropout immediately after completion of the course (Breier *et al*. 2009:80, 124).

Evidence of the possibility of transforming the negative perceptions of nursing amongst nursing students by means of a variety of interventions was reported in a number of studies. It was found that after exposure to theory and a variety of practitioners (positive professional role models) in clinical settings during the 4 year basic training, student nurses’ image of nursing changed from a lay to a professional image and from idealistic to a more realistic one. In addition, it was found that professionalism was enhanced amongst student nurses through exposure to positive clinical experiences (Day *et al.* 2005:640; Safadi *et al*. 2011:4; Valizadeh *et al.* 2008:1085). Even though Bolan and Grainger (2009:775) found that some of the students maintained some traditional and idealistic beliefs from year one to year four of training, they also found that it was possible to transform the negative perceptions of nursing that still persisted amongst student nurses before the end of training, and to retain the desired positive perceptions gained through the years of training.

## Problem statement

The evidence indicates that a positive image of nursing attracts applicants into the nursing profession (Seago *et al.* 2006). It also signifies that the intention to leave nursing correlates positively with holding a negative image of nursing (Varaei *et al.* 2012:551). There is a need thus for the profession and nurse educators to develop and use innovative strategies to enhance the image of nursing and to transform the negative image of nursing amongst student nurses. Appreciative inquiry (AI) was proposed as an intervention teaching strategy to transform student nurses’ image of nursing.

## Significance of the study

A literature search on the use of AI to advance new learning and teaching approaches that are pertinently designed to change perceptions and promote appreciation of nursing amongst student nurses yielded no results. The findings of the study have the potential to contribute to the body of knowledge of nursing education regarding innovative teaching strategies that are pertinently designed to promote a professional image and appreciation of nursing amongst student nurses.

Research has shown that nursing students begin their nursing education with stereotypical, idealized and inaccurate images of nursing that change over years of education and training (Day *et al.* 2005:4; Emeghebo 2012:7; Safadi *et al.* 2011:4), but most of the student nurses maintain some traditional and idealistic beliefs from year one to year four of training (Bolan & Grainger 2009:775). The importance of focusing on transforming the image of nursing amongst the third and fourth year student nurses was to address the negative perceptions of nursing that persisted amongst student nurses before the end of training and to retain the desired perceptions gained through the years of training.

## Study purpose

The purpose of the study was to explore the potential of AI as an intervention teaching strategy to transform student nurses’ image of nursing.

## Research design and methods

### Design

A quantitative, quasi-experimental explorative-descriptive design comprising the pretest, AI as intervention, and the post-test was utilised.

### Population and sampling

The study population comprised student nurses registered for the programme of education and training leading to registration as a nurse (general, psychiatric and community) and midwife (South Africa 1985: paragraph [iii] as amended). Convenience sampling was used to select third and fourth year male and female student nurses from one nursing college, and nursing departments of the two selected universities in the City of Tshwane, Gauteng province, South Africa who were registered for the diploma and bachelors’ degree in nursing respectively for the 2012 academic year.

### Data collection

One self-administered questionnaire was used to collect data on the student nurses’ perceptions of the image of nursing before and after the application of AI.

A four-point Likert-type scale was used to rate the responses using the response categories: strongly agree, agree, disagree and strongly disagree. The 63 questionnaire items were grouped into the subscales: the nature of nursing work, nursing as a career, characteristics and qualifications required for entry, working conditions, perceptions about nurses, gender in nursing and the meaningfulness of nursing.

### Pretest

The pre-intervention survey was conducted using a convenience sample of 220 student nurses. The aims of the pretest were to establish and describe the baseline perceptions of the image of nursing amongst student nurses and to guide the purposive selection of participants for AI ntervention.

### Intervention

AI was introduced as an intervention with the aim of positively changing the image of nursing amongst student nurses. Based on the pretest results, 28 student nurses were selected for participation in 2 AI interview sessions of three hours duration each. The selected student nurses who participated in AI consisted of 12 student nurses at the top of the scale of the questionnaire (Group A) and 16 students at the bottom of the scale of the questionnaire. Selecting participants from the top and the bottom of the scale of the questionnaire provided a broad range of participants who fell outside of the average variability of scores (Average + Standard Deviation [SD]) in the pretest.

### Appreciative inquiry

AI is a philosophy and method for promoting transformational change, shifting from a traditional problem-based orientation to a strength-based approach to change that focuses on affirmation, appreciation and positive dialogue (Trajkovski *et al.* 2013:95). Instead of focusing on deficiencies and problems, AI focuses on existing organisational strengths rather than weaknesses and on discovering what works best, why it works and how success can be expanded in the organisation (Bushe 2007; Elleven 2007:451; Kavanagh *et al.* 2010:2). AI is based on the constructionist perspective on social reality and on the belief that human systems move in the direction of their shared image and idea of the future (common vision). According to Chapagain and Ojha (2008), human actions are prescribed by ideas, beliefs, intentions, interests, purposes and means, values, habits and theories. However, the transformation of human behaviour is achieved by changing conventional ideas, values, beliefs, intentions, and means.

In the study, AI encompassed the 4-D cycle: Discovery (positive elements of the image of nursing illuminated), Dream (the ideal image of nursing envisioned), Design (processes were created to support the ideal envisioned image of nursing) and Destiny (strategies that strive for the ideal image of nursing implemented). The 4-D cycle of AI enabled student nurses to describe their peak nursing experiences, envision their shared and desired future image of nursing and generate statements about the image of nursing they want to achieve (provocative propositions). The AI process led to the development of specific action plans aimed at achieving the ideal future image of nursing and implementing the designed lans.

### Post-test

A total of 28 eligible student nurses participated in the post-test. The eligibility criterion for the post–test was participation in the intervention because the aim was to determine whether AI produced a positive nursing image change and positive orientation towards nursing amongst student nurses. Data on student nurses’ post-appreciative inquiry perceptions of the image were collected by the same questionnaire that was used in the pretest. All the 28 student nurses returned the completed questionnaire, resulting in a response rate of 100%.

### Validity and reliability of the instrument

The questionnaire was developed following a thorough study of the relevant literature and was pretested on a convenience sample of 15 student nurses, none of whom participated in the actual study (Polit & Beck 2012:723). Two nurse leaders and two nurse educators were requested to review the questionnaire in order to determine the content and construct validity as measured against standard professional codes, caring ethics and appreciation of nursing in general. The face and content validity were acceptable according to the feedback from the consulted statistical and content experts. The questionnaire items showed a good content validity index of 0.90 (Polit, Beck & Owen 2006). The Cronbach's alpha reliability coefficient was used as an estimate of the internal consistency of the whole questionnaire, which was deemed acceptable at 0.77. According to Rowe, Dancey and Reidy (2012:337), a Cronbach's alpha of > 0.70 is sufficient for research instruments to be considered reliable.

### Data analysis

Data analysis was performed by means of SPSS version 20.0 using the same descriptive statistics for the pre- and post-test. In addition, McNemar's tests in SPSS were used to detect the differences in student nurses’ pre- and post-test responses to questionnaire items. The *p*-value < 0.05 indicated statistically significant differences between the pre- and the post-test results (Adedokun & Burgess 2012).

## Results

### Biographical data

The results of the biographical data are shown in Table 1.

**TABLE 1:** Pretest-post-test biographical data.

Variables	Pretest (*N* = 220)			Post-test (*N* = 28)		
	Diploma in nursing (%)	Bachelor's degree (%)	Total (%)	Diploma in nursing (%)	Bachelor's degree (%)	Total (%)
**Gender**						
Male	18 (8.2)	14 (6.4)	32 (14.5)	5 (17.9)	2 (7.1)	7 (25)
Female	127 (57.7)	61 (27.7)	188 (85.5)	10 (35.7)	11 (39.3)	21 (75)
**Age in years**						
18–20	7 (3.2)	4 (1.8)	11 (5.0)	-	-	-
21–23	98 (44.5)	43 (19.5)	141 (64.0)	11 (39.2)	5 (17.9)	16 (57.1)
24–26	40 (18.2)	28 (12.7)	68 (31.0)	4 (14.3)	8 (28.6)	12 (42.9)
27 >	-	-	-	-	-	-
**SANC registration**						
Yes	145 (65.9)	75 (34.1)	220 (100)	15 (53.6)	13 (46.4)	28 (100)
No	-	-	-	-	-	-
**Level of training**						
3rd year	106 (48.2)	61 (27.7)	167 (75.9)	10 (35.7)	11 (39.3.1)	21 (75)
4th year	39 (17.2)	14 (6.9)	53 (24.1)	5 (17.9)	2 (7.1)	71 (25)

The findings show that the student nurses who took both tests were predominantly female with the majority being students from the nursing college and students in the third year of training. The mean age was 22 years (SD 3.00) in the pretest and in the post-test it was 23 years (SD 1.12).

The results for group A show a statistically significant increase of 16% (McNemar test *p*-value = 0.002) in the number of agreement ratings for item 2.1 *(nursing is a respected profession*) from the pretest 42% to the post-test 58%, and an increase of 50% (McNemar test *p*-value = 0.001) in the number of agreement ratings for item 2.3 (*nursing is an appreciated profession*) from the pretest 25% to the post-test 75%. In group B, increases of 62.6% (McNemar test *p*-value = 0.018) n the number of agreement ratings from the pretest 18.6% to the post-test.

**TABLE 2:** Student nurses’ perceptions of nursing as a profession before and after AI.

The Nature of Nursing	Participants: Nursing is	Pretest / Post-test Responses (*N* = 28)					
		Group A (*n* = 12)			Group B (*n* = 16)		
		Pre-AI *n* (%)	Post-AI *n* (%)	*p*-value	Pre-AI *n* (%)	Post-AI *n* (%)	*p*-value
Nursing is a respected profession	Strongly agree/agree	5 (42)	7 (58)	0.002*	3 (18.6)	13 (81.2)	0.018*
	Strongly disagree/disagree	7 (58)	5 (42)		14 (87.5)	3 (18.6)	
Nursing is based on helping others	Strongly agree/agree	10 (85)	11 (92)	0.180	13 (81)	13 (81)	1.00
	Strongly disagree/disagree	2 (15)	1 (8)		3 (19)	3 (19)	
Nursing is an appreciated profession	Strongly agree/agree	3 (25)	9 (75)	0.001*	5 (31.2)	11 (68.8)	0.001*
	Strongly disagree/disagree	9 (75)	3 (25)		11 (68.8)	5 (31.2)	
Nursing is an independent profession	Strongly agree/agree	7 (59)	9 (71)	0.152	14 (87.5)	15 (93.7)	0.180
	Strongly disagree/disagree	5 (41)	3 (29)		2 (12.5)	1 (6.3)	
Nursing is a prestigious profession	Strongly agree/agree	8 (65)	9 (75)	0.210	10 (65)	12 (75)	0.210
	Strongly disagree/disagree	4 (35)	3 (25)		6 (35)	4 (25)	
Nursing is an indispensable profession in any society	Strongly agree/agree	10 (85)	11 (92)	0.18	13 (81)	13 (81)	1.00
	Strongly disagree/disagree	2 (15)	1 (8)		3 (19)	3 (19)	
Nursing should be a university programme	Strongly agree	9 (75)	11 (91.6)	0.001*	2 (12.5)	14 (87.5)	0.018*
	Strongly disagree/disagree	3 (25)	1 (8.4)		14 (25)	2 (12.5)	
Nursing is a good career choice for students with good grades	Strongly agree/agree	10 (85)	11 (92)	0.180	9 (59)	11 (71)	0.152
	Strongly disagree/disagree	2 (15)	1 (8)		7 (41)	5 (29)	

AI, appreciative inquiry.

*, McNemar's test; statistically significant *p*-value < 0.05

Increases to 81.2% for item 2.1 and 37.6% (McNemar test *p*-value = 0.001) from the pretest 31.2% to the post-test 68.8% for item 2.3 were observed. The implication of this is that the student nurses’ negative perceptions of nursing with regard to respect and appreciation had changed and became positive following the implementation of AI.

**TABLE 3:** Student nurses’ perceptions about nurses before and after appreciative inquiry intervention.

Student Nurses’ Perceptions about Nurses	Participants	Pretest –post-test Responses (*N* = 28)					
		Group A (*n* = 12)			Group B (*n* = 16)		
		Pre-AI *n* (%)	Post-AI *n* (%)	*p*-value	Pre-AI *n* (%)	Post-AI *n* (%)	*p*-value
**Nurses will always have a job**							
	Strongly agree/agree	10 (85)	11 (92)	0.180	13 (81)	13 (81)	1.00
	Strongly disagree/disagree	2 (15)	1 (8)		3 (19)	3 (19)	
**Nurses need good grades**							
	Strongly agree/agree	8 (65)	9 (75)	0.210	9 (59)	11 (71)	0.152
	Strongly disagree/disagree	4 (35)	3 (25)		7 (41)	5 (29)	
**Nurses are appreciated**							
	Strongly agree/agree	5 (42)	7 (58)	0.002*	3 (18.6)	13 (81.2)	0.018*
	Strongly disagree/disagree	7 (58)	5 (42)		14 (87.5)	3 (18.6)	
**Nurses are respected**							
	Strongly agree/agree	3 (25)	9 (75)	0.001*	5 (31.2)	11 (68.8)	0.001*
	Strongly disagree/disagree	9 (75)	3 (25)		12 (75)	4 (25)	
**Nurses are recognised enough for their contribution**							
	Strongly agree/agree	10 (85)	11 (92)	0.180	13 (81)	13 (81)	1.00
	Strongly disagree/disagree	2 (15)	1 (8)		3 (19)	3 (19)	
**Nurses are caring people**							
	Strongly agree/agree	11 (91.9)	12 (100)	0.687	10 (65)	12 (75)	0.210
	Strongly disagree/disagree	1 (8.1)	0 (0)		6 (35)	4 (25)	
**Nurses are independent thinkers**							
	Strongly agree/agree	10 (85)	11 (92)	0.180	13 (81)	13 (81)	1.00
	Strongly disagree/disagree	2 (15)	1 (8)		3 (19)	3 (19)	

AI, appreciative inquiry.

*, McNemar's test; statistically significant *p*-value *<* 0.05

A statistically significant increase of 16.6% (McNemar test *p*-value = 0.001) in the number of agreement ratings for item 2.7 (*nursing should be a university programme*) from the pretest 75% to the post-test 91.6% was observed in group A, whilst in group B an increase of 75% from the pretest 12.5% to the post-test 87.5% was statistically significant for item 2.7. The increases suggest a more positive perception of the entry requirements for nursing as a result of the AI intervention. Although there were increases in the number of agreement ratings in other items of this subscale, those increases were statistically non-significant (McNemar test *p*-value = 0. 0.180 for item 2.4 and 0.210 for item 2.5).

Statistically significant differences in the pretest/post-test responses for the items 3.3 (*nurses are appreciated*) and 3.4 (*nurses are respected*) were observed in group A and group B. In group A, there were statistically significant increases of 16% (McNemar test *p*-value = 0.002) in the number of agreement ratings for item 3.3 from the pretest 42% to the post-test 58%, and an increase of 50% (McNemar test *p*-value = 0.001) in the number of agreement ratings for item 3.4 from the pretest 25% to the post-test 75%. In group B, increases of 62.6% (McNemar test *p*-value = 0.018) in the number of agreement ratings from the pretest 18.6% to the post-test 81.2% for item 3.3 and 37.6% (McNemar test *p*-value = 0.001) from the pretest 31.2% to the post-test 68.8% for item 3.4 were statistically significant. The implication is that the student nurses’ negative perceptions about nurses had changed and became positive following the intervention.

The pretest/post-test responses to four items of the subscale *student nurses’ perceptions of gender in nursing* for group A and group B are shown in Table 4. The post-test results showed an increase in the number of agreement ratings for item 4.1 (*Males are as good nurses as females*) for both groups A and B. The increase in the number of agreement ratings for group A was not statistically significant, whilst the increase for group B was statistically significant (McNemar test *p*-value < 0.05). The findings for other items in this subscale were statistically non-significant. The post-test results also showed statistically insignificant increases in the number of disagreement ratings for items 4.2, 4.3 and 4.4 for both groups A and B.

According to the results summarized in Table 5, there was a statistically significant increase of 16.6% (McNemar test *p*-value = 0.001) in the number of students who disagreed with item 5.1 (*Nurses work in a safe place*) from the pretest 75% to the post-test 91.6% in group A. In group B, a 12.5% (McNemar test *p*-value = 0.001) increase in the number of student nurses who disagreed with the same item was observed; from the pretest 75% to the post-test 87.5%. This increase was statistically significant (*p*-value < 0.05). In the pretest, item 5.1 obtained disagreement ratings (negative perception) which increased significantly in the post-test for both group A and group B, suggesting that the negative perception remained and became more negative in the post-test.

**TABLE 4:** Student nurses’ perceptions of gender in nursing before and after appreciative inquiry intervention.

Gender in Nursing	Participants responses (Extreme Cases)	Pretest/Post/Test Responses (*N* = 28)					
		Group A (*n* = 12)			Group B (*n* = 16)		
		Pre-AI *n* (%)	Post-AI *n* (%)	*p*-value	Pre-AI *n* (%)	Post-AI *n* (%)	*p*-value
**Males are as good nurses as females**							
	Strongly agree/agree	11 (91.6)	12 (100)	0.581	8 (50)	14 (87.5)	0.020*
	Strongly disagree/disagree	1 (8.4)	0 (0)		8 (50)	2 (12.5)	
**Male nurses are more accepted by patients than female nurses**							
	Strongly agree/agree	4 (35)	3 (25)	0.21	2 (12.5)	1 (6.3)	0.18
	Strongly disagree/disagree	8 (65)	9 (75)		14 (87.5)	15 (93.7)	
**Doctors prefer male nurses more than female nurses**							
	Strongly agree/agree	4 (35)	3 (25)	0.210	7 (41)	5 (29)	0.152
	Strongly disagree/disagree	8 (65)	9 (75)		9 (59)	11 (71)	
**Male nurses are not respected by others**							
	Strongly agree/agree	1 (8.4)	0 (0)	0.581	7 (41)	5 (29)	0.152
	Strongly disagree/disagree	11 (91.6)	12 (100)		9 (59)	11 (71)	

AI, appreciative inquiry.

*, McNemar's test; statistically significant *p*-value < 0.05

**TABLE 5:** Student nurses’ perceptions of the working conditions of nurses before and after AI.

Working Conditions	Participants responses	Pretest/Post-test Responses (*N* = 28)					
		Group A (*n* = 12)			Group B (*n* = 16)		
		Pre-AI *n* (%)	Post-AI *n* (%)	*p*-value	Pre-AI *n* (%)	Post-AI *n* (%)	*p*-value
**Nurses work in a safe place**							
	Strongly agree	3 (25)	1 (8.4)	0.001*	4 (25)	2 (12.5)	0.001*
	Strongly disagree/disagree	9 (75)	11 (91.6)		12 (75)	14 (87.5)	
**Nurses work with high technology**							
	Strongly agree/agree	8 (65)	9 (75)	0.210	9 (59)	11 (71)	0.152
	Strongly disagree/disagree	4 (35)	3 (25)		7 (41)	5 (29)	
**Nurses make/earn a lot of money**							
	Strongly agree/agree	7 (58)	5 (42)	0.002*	14 (25)	2 (12.5)	0.018*
	Strongly disagree/disagree	5 (42)	7 (58)		2 (12.5)	14 (87.5)	
**Nurses are major players within the high tech medical world**							
	Strongly agree/agree	11 (91.6)	12 (100)	0.581	9 (59)	11 (71)	0.152
	Strongly disagree/disagree	1 (8.4)	0 (0)		7 (41)	5 (29)	

AI, appreciative inquiry.

*, McNemar's test; statistically significant *p*-value < 0.05

With regard to item 5.3 (*Nurses make/earn a lot of money*), a statistically significant increase of 16% (McNemar test *p*-value = 0.002) in the number of students who disagreed with item 5.3 from the pretest 42% to the post-test 58% was observed in group A, whilst in group B an increase of 75% from the pretest 12.5% to the post-test 87.5% was statistically significant for this item. Consequently the number of student nurses who disagreed that nurses make or earn a lot of money increased in both groups A and B.

The results of both the pre- and post-test showed no statistically significant differences in the pretest and/or post-test responses to each item of the subscales *nursing as a career* and *meaningfulness of nursing* following the implementation of AI for both group A and B. The implication was that the perceptions of the image of nursing as a career and its meaningfulness had not changed in the post-test; they remained positive following AI intervention.

## Discussion of results

The female student nurses represented in the study reflected the gender distribution in the nursing profession, where female student nurses are in the majority. According to the South African Nursing Council (SANC) (2008) records, the ratio of male to female student nurses increased from 1:5 in 2003 to 1:3 in 2007, showing that nursing remains a female dominated profession. Seago *et al.* (2006:96) emphasise that the perception of nursing as a woman's occupation gives it a subordinate occupation status. Furthermore, Breier *et al*. (2009:127) point out that the professions attracting a significant number of women, or a majority of women, lose status and earning power because the society associates women with weakness, dependence and powerlessness (Neilson & Lauder 2008:688). The findings were also reflective of the distribution of nursing students at SA nursing education institutions where nursing college student nurses are in the majority. The nursing colleges enrol more student nurses for the programme of education and training leading to registration as a nurse (general, psychiatric and community) and a midwife (Regulation R.425, 1985 as amended) than the universities.

The pretest results revealed a mix of positive and negative perceptions of the image of nursing amongst student nurses. According to the pretest results, nursing was perceived negatively as a profession that is not respected and appreciated and nurses as professionals who were not respected, appreciated and recognised for their contribution. Within the SA literature, lack of appreciation and respect for nursing within the profession was reported (Breier *et al.* 2009; Mkhize & Nzimande 2007). In addition, the finding that the majority of student nurses perceived nursing negatively as work done in unsafe working conditions with inadequate remuneration was not surprising because similar perceptions amongst SA nurses were reported in the Nursing Compact (South Africa 2012) and the National Strategic Plan for Nurse Education, Training and Practice (South Africa 2012).

The positive baseline (pretest) perceptions of the image of nursing included the student nurses’ perceptions about the entry requirements for nursing, men in nursing, nursing as a career as well as meaningfulness of nursing. These results are in line with one of the study assumptions that a positive core of experiences exists amongst student nurses. The results are also supported by the assumption of AI that in every organisation, something works right.

The intervention, carried out according to the principles of AI was intended to change the student nurses’ negative perceptions of the image of nursing. The post-test results showed a significant and positive change in the student nurses’ perception of the image of nursing as a respected and appreciated profession and nurses as respected and appreciated professionals. San Martin and Calabrese (2011:116) conducted a qualitative case study to facilitate the discovery and dream phases of AI in order to empower ‘at-risk’ students. The results of the same study revealed that the core values of appreciation and respect became important parts of ‘at-risk’ students’ envisioned future image of nursing as it occurred in this study.

AI did not merely change the student nurses’ negative perceptions of nursing in a positive direction, but some of the positive and negative perceptions were enhanced. The baseline positive perceptions that were enhanced as a result of the use of AI were student nurses’ perceptions of the entry requirements for nursing and men in nursing. Similar results were obtained in Cojocaru and Brăgaru’s (2012) study which used AI to change perceptions concerning the satisfaction of members’ need for security. The results of that study showed that, although AI was directed mainly towards changing the perceptions regarding the satisfaction of the need for security, the interpretations given by organisation members changed with regard to the satisfaction of all needs (security, basic needs, belonging, esteem and self-actualisation). The student nurses’ baseline perceptions of the working conditions of nurses did not change from negative to positive; instead they remained and became more negative as a result of AI. The possible explanation for the negative perception of the nurses’ working conditions before and after AI relates to the newly acquired, positive post-appreciative inquiry perception of the image of nursing that the student nurses felt that nurses were entitled to work in safe environments with adequate human and financial resources. There were no statistically significant changes in the student nurses’ positive perceptions of nursing as a career and meaningfulness of nursing after the implementation of AI.

## Ethical considerations

Ethical clearance was granted by the higher degrees committee of the Department of Health Studies, University of South Africa. Permission to conduct the study was obtained from the Gauteng Provincial Department of Health, the management of nursing departments of the selected universities and the management of the selected nursing college.

The participants gave consent after they had been informed about the purpose, the nature, the process and the activities of the study. They were not required to give a written consent; response to the questionnaires implied voluntary informed consent. Confidentiality and anonymity were assured because only the pseudonyms were written on the questionnaire. The raw data were kept confidential, locked up with no unauthorised access. Data were reported in a manner that did not identify or link the participants with the information. No remuneration was paid.

### Practical implications

The study suggests the use of AI as a teaching strategy to transform the image of nursing and to promote a positive professional image of nursing amongst student nurses. The principles and assumptions of AI support collaborative learning, active participation by all members (as individuals, in pairs and in groups) and learning from the ‘positive’ known to the unknown.

Further research focusing on a broader population needs to be conducted using the same research methodology as this study. The need for studies evaluating the sustainability of post-AI perceptions on a long-term basis has been identified.

## Limitations

Quantitative results were specific to the third and fourth year student nurses at university nursing departments and a nursing college in the City of Tshwane, Gauteng province. Consequently, the survey results could not be generalised to third and fourth year student nurses in other provinces of South Africa as well as first and second year student nurses in other nursing colleges and university nursing departments in the Gauteng and other provinces.

## Conclusion

The findings of the pretest highlighted aspects of the image of nursing that were perceived positively and negatively by student nurses. The relevance of this finding for nurse educators is the importance of continual assessment of the perceptions of the image of nursing amongst student nurses and appropriate planning of strategies pertinently focusing on the promotion of the positive image of nursing amongst student nurses.

The post-intervention survey results showed that some of the aspects of the student nurses’ image of nursing, such as perceptions about nurses and nursing as a profession, changed significantly and in a positive direction. Another noteworthy finding was that AI did not merely result in a change from negative to positive perceptions in some aspects of student nurses ‘image of nursing, but the negative perceptions of the working conditions of nurses remained and became more negative following the implementation of AI. According to the findings of the study, AI has shown potential as a teaching strategy to transform the student nurses’ image of nursing.
